# Five-year follow-up of nivolumab treatment in Japanese patients with esophageal squamous-cell carcinoma (ATTRACTION-1/ONO-4538-07)

**DOI:** 10.1007/s10388-021-00850-0

**Published:** 2021-05-16

**Authors:** Taroh Satoh, Ken Kato, Takashi Ura, Yasuo Hamamoto, Takashi Kojima, Takahiro Tsushima, Shuichi Hironaka, Hiroki Hara, Satoru Iwasa, Kei Muro, Hirofumi Yasui, Keiko Minashi, Kensei Yamaguchi, Atsushi Ohtsu, Yuichiro Doki, Yasuhiro Matsumura, Yuko Kitagawa

**Affiliations:** 1 Department of Frontier Science for Cancer and Chemotherapy, Osaka University Graduate School of Medicine, E21-19, 2-2, Yamadaoka, Suita, Osaka 565-0871 Japan; 2 Department of Head and Neck, Esophageal Medical Oncology / Department of Gastrointestinal Medical Oncology, National Cancer Center Hospital, Tokyo, Japan; 3 Department of Clinical Oncology, Aichi Cancer Center Hospital, Nagoya, Japan; 4 Department of Clinical Oncology, National Hospital Organization Kyoto Medical Center, Kyoto, Japan; 5 Keio Cancer Center, Keio University School of Medicine, Tokyo, Japan; 6 Department of Gastroenterology and Gastrointestinal Oncology, National Cancer Center Hospital East, Kashiwa, Japan; 7 Division of Gastrointestinal Oncology, Shizuoka Cancer Center, Shizuoka, Japan; 8 Clinical Trial Promotion Department, Chiba Cancer Center, Chiba, Japan; 9 Department of Medical Oncology and Hematology, Oita University Faculty of Medicine, Oita, Japan; 10 Department of Gastroenterology, Saitama Cancer Center, Saitama, Japan; 11 Department of Gastroenterological Chemotherapy, The Cancer Institute Hospital of Japanese Foundation for Cancer Research, Tokyo, Japan; 12 National Cancer Center Hospital East, Kashiwa, Japan; 13 Department of Gastroenterological Surgery, Osaka University Graduate School of Medicine, Osaka, Japan; 14 Department of Oncology, ONO Pharmaceutical Co., Ltd., Osaka, Japan; 15 Department of Surgery, Keio University School of Medicine, Tokyo, Japan

**Keywords:** Nivolumab, Esophageal squamous cell carcinoma, Clinical trial, Immunotherapy

## Abstract

**Background:**

In the phase II ATTRACTION-1 study, nivolumab demonstrated a promising antitumor activity among Japanese patients with treatment-refractory advanced esophageal cancer. Here, we report the follow-up results of ATTRACTION-1 of > 5 years.

**Methods:**

We enrolled patients with esophageal cancer that was refractory or intolerant to a standard chemotherapy. Then, nivolumab (3 mg/kg) was administered every 2 weeks. The primary endpoint was a centrally assessed objective response rate.

**Results:**

Nivolumab was administered to 65 patients with esophageal squamous-cell carcinoma (ESCC). The centrally assessed objective response rate was 17.2%. The overall survival rates at 3 and 5 years were 10.9% and 6.3%, respectively. Three-year survivors tended to have more reduced target lesions. A total of 63.1% of the patients exhibited treatment-related adverse events, and no new safety signal was observed. Patients with select adverse events tended to have better overall survival than those without. No apparent chronological order was observed between the first response and the onset of select adverse events.

**Conclusion:**

Our follow-up analysis of more than 5 years is currently the longest and is the first to demonstrate that nivolumab has long-term efficacy and safety for advanced ESCC.

**Supplementary Information:**

The online version contains supplementary material available at 10.1007/s10388-021-00850-0.

## Introduction

Esophageal cancer is one of the most common malignancies, with over 500,000 cases of new diagnosis and death worldwide in 2020 [1]. Monotherapy of docetaxel or paclitaxel has been applied to patients with esophageal cancer that are refractory or intolerant to fluoropyrimidine plus platinum-based chemotherapy; however, these alternatives have generally poor long-term outcomes [2–4]. In addition, only few treatment options for second- or later-line therapy are available.

Immune checkpoint inhibitors, including nivolumab, have provided major breakthroughs in cancer therapy [5]. In particular, long-term survival with durable responses is a remarkable feature of treatment with immune checkpoint inhibitors [6–12]. Nivolumab, which is an anti-PD-1 antibody, inhibits interaction between PD-1 and its ligands, such as PD-L1, thereby facilitating the antitumor activity of T lymphocyte [13]. In the Japanese phase II ATTRACTION-1 study, nivolumab exhibited a promising antitumor activity in patients with esophageal cancer that was refractory or intolerant to a standard chemotherapy [14]. In the subsequent phase III ATTRACTION-3 study, nivolumab significantly improved the overall survival (OS) compared with chemotherapy as a second-line therapy for esophageal cancer [15]. Through these findings, nivolumab was approved in Japan in February 2020, for esophageal cancer that is refractory or intolerant to a standard chemotherapy, and nivolumab was further approved as a second-line treatment for esophageal cancer in South Korea, the United States, Taiwan, Brazil, and the European Union.

We previously reported 2-year follow-up results of ATTRACTION-1 [16]. Here, we report the longest follow-up data of nivolumab monotherapy for esophageal cancer for at least 5 years in ATTRACTION-1.

## Methods

### Study design and patients

This study conformed to the Declaration of Helsinki and Good Clinical Practice guidelines. All patients provided written informed consent. The study protocol was approved by the institutional review board at each study site.

ATTRACTION-1 is an open-label, single-arm, multicenter, phase II study conducted in Japan. Detailed methods of ATTRACTION-1 were described elsewhere [14]. In brief, patients who had esophageal cancer in the cervical or thoracic esophagus and were refractory or intolerant to fluoropyrimidine-based, platinum-based, and taxane-based chemotherapy were enrolled between February 25, 2014 and November 14, 2014. Other inclusion criteria included the presence of at least one measurable lesion that was unsuitable for radical resection, an Eastern Cooperative Oncology Group performance status of 0 or 1, and adequate organ function.

### Procedures

Patients intravenously received 3 mg/kg of nivolumab every 2 weeks in 6-week cycles until the disease progression or unacceptable toxicity. Computed tomography of the neck, chest, abdomen, and pelvis was performed every 6 weeks in the first 8 cycles and every 12 weeks thereafter. We assessed the antitumor activity according to the Response Evaluation Criteria in Solid Tumors version 1.1, which includes complete response (CR), partial response (PR), stable disease, progressive disease (PD), and not assessable. The Japan Clinical Oncology Group translation of the Common Terminology Criteria for Adverse Events version 4.0 was used for grading adverse events (AEs).

### Endpoints

The primary endpoint was the centrally assessed objective response rate (ORR), which was defined as the proportion of patients who achieved CR or PR. The secondary endpoints were the OS, progression-free survival (PFS), maximum change in tumor burden, best overall response (BOR), and others.

### Statistical analysis

After nivolumab was approved for esophageal cancer in Japan on February 21, 2020, administration of nivolumab as the study drug in ATTRACTION-1 discontinued and commercially available nivolumab was used. For efficacy endpoints, this study used the data as of March 5, 2020, which was the last day of efficacy evaluation, and safety analyses used the data as of June 12, 2020, which was the time of the final data cutoff. Patients who received at least one dose of nivolumab were assessed for safety endpoints, and those with at least one central assessment of tumor response were assessed for efficacy endpoints. OS and PFS were analyzed by the Kaplan–Meier method with estimated median values and their 95% confidence intervals (CIs) calculated with normal approximation. In a post hoc analysis, OS according to experience of select AEs (Supplementary Table 1) for the whole study period was assessed by the Kaplan–Meier method. All statistical data were analyzed using SAS version 9.4 (SAS Institute Inc., Cary, NC, USA).

## Results

### Patients and treatments

ATTRACTION-1 enrolled 65 patients with esophageal squamous-cell carcinoma (ESCC). All 65 patients were assessed for safety; however, one patient had multiple primary tumor sites, thereby excluded from the efficacy analysis. Patient characteristics at baseline were described previously [14]. All patients had ESCC, and 21 (32.3%) and 20 (30.8%) patients received ≤ 2 and ≥ 4 prior systemic therapies, respectively.

At the approval date of nivolumab for ESCC in Japan, two patients were on the study treatment.

### Efficacy

**Fig. 1:**
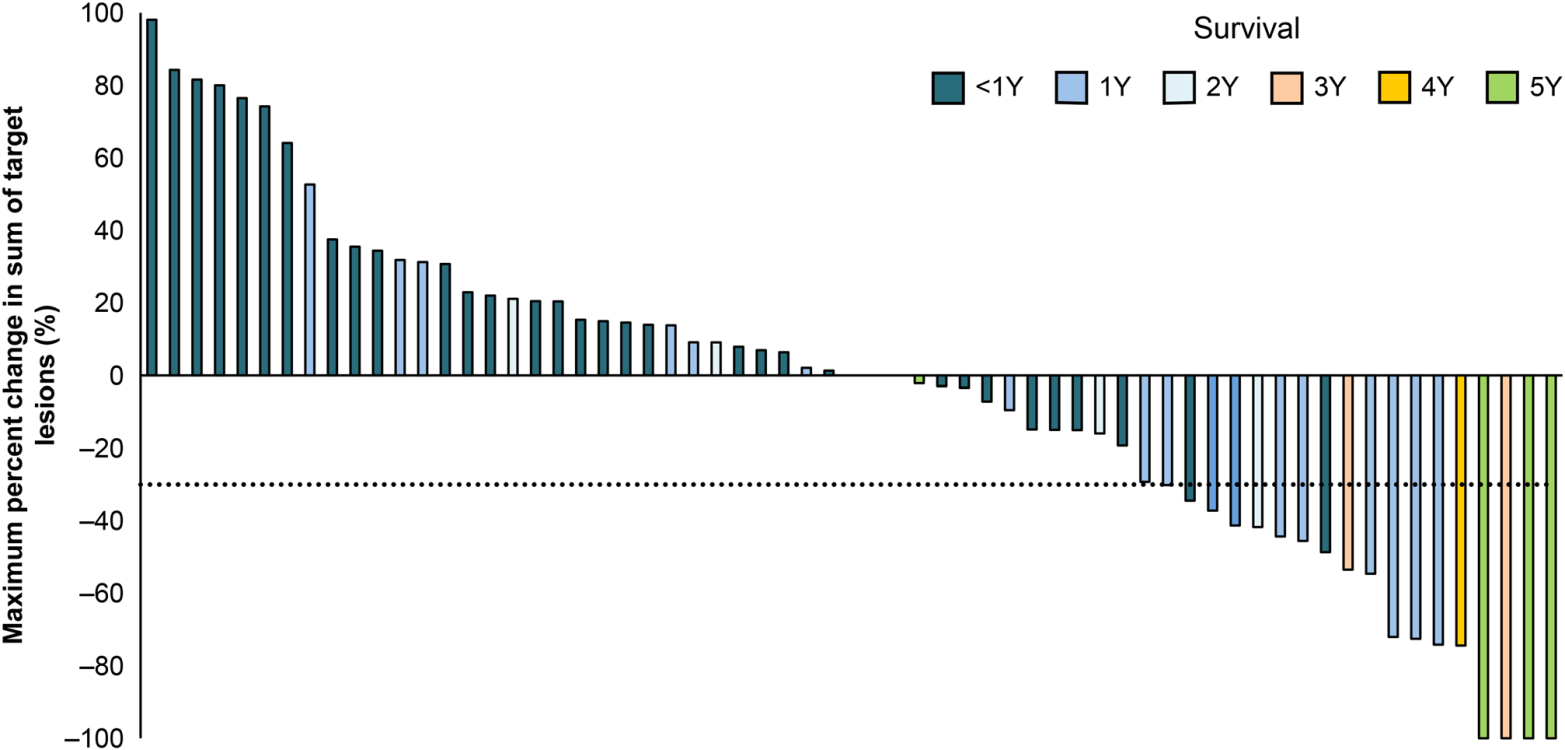
Change of target lesion sizes. The waterfall plot highlights maximum percent change in the sum of target lesion size from baseline that were assessed by each investigator. Each color represents patients surviving for < 1 (navy blue), 1 (blue), 2 (cyan), 3 (pink), 4 (orange), and 5 (green) years

The centrally assessed ORR was 17.2%, and three patients achieved CR (Supplementary Table 2). In patients achieving CR or PR, the median time to initial response was 1.4 months, and the median duration of response was 11.2 months. Through investigator assessment, target lesions were found to be reduced in approximately half of the patients (Fig. 1).

**Fig. 2:**
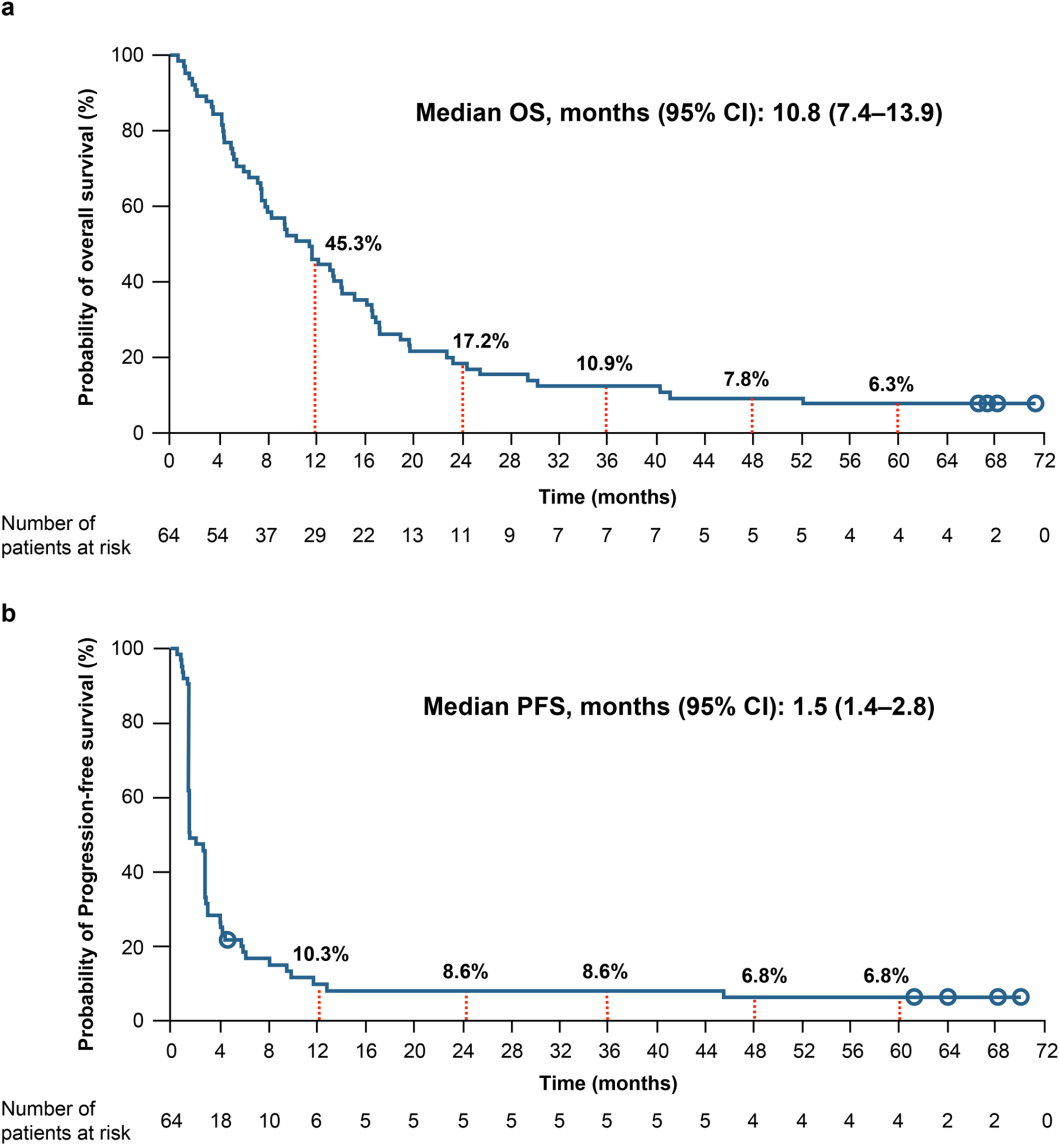
Probability of survival. The Kaplan–Meier curves show the **a** overall survival (OS) and **b** progression-free survival (PFS). Circles represent censors. *CI*, Confidence interval

The median OS was 10.8 months, and 3-, 4-, and 5-year OS rates were 10.9%, 7.8%, and 6.3%, respectively (Fig. 2a). Seven patients survived for more than 3 years, while four patients survived for more than 5 years. Meanwhile, the median PFS was 1.5 months, and the Kaplan–Meier curve of PFS reached a plateau with 3-, 4-, and 5-year PFS rates of 8.6%, 6.8%, and 6.8%, respectively (Fig. 2b).

### Long-term survivors

Table 1 summarizes the BOR in patients stratified by survival periods. All of the three patients with CR survived more than 5 years (Table 1). In addition, target lesions tended to reduce greatly in these long-term survivors (Fig. 1).

**Table 1:** Best overall response of long-term survivors

Best overall response (central review)	All patients (*N* = 64)	2-year survivors (*N* = 11)	3-year survivors (*N* = 7)	5-year survivors (*N* = 4)
Complete response	3 (4.7)	3 (27.3)	3 (42.9)	3 (75.0)
Partial response	8 (12.5)	3 (27.3)	2 (28.6)	0
Stable disease	16 (25.0)	3 (27.3)	1 (14.3)	0
Progressive disease	29 (45.3)	1 (9.1)	0	0
Not assessable	8* (12.5)	1 (9.1)	1 (14.3)	1 (25.0)

*Includes patients with no target lesions

The characteristics of patients who survived more than 3 years are shown in Table 2. These 3-year survivors included patients with stage IV disease, comprehensive pretreatment, and negative PD-L1 expression on tumors.

**Table 2:** Characteristics of 3-year survivors

	Patient 1	Patient 2	Patient 3	Patient 4	Patient 5	Patient 6	Patient 7
Sex	Male	Female	Male	Male	Female	Male	Male
Age	64	64	80	68	67	57	57
ECOG PS	1	0	1	0	1	0	0
Alcohol consumption (habitual)	Former	Former	Former	Current	Current	Current	Former
Smoking status	Former	Former	Former	Former	Former	Former	Former
Prior radiation therapy	No	Yes	Yes	No	Yes	Yes	No
Prior surgery	Yes	No	Yes	Yes	No	Yes	Yes
Number of prior systemic therapy	4	2	3	2	3	3	6
Disease stage	IIB	IIIC	IV	IIB	IA	IV	unknown
Size of the target lesion (mm)	111	91.02	15.04	43.5	15.28	17.35	12.38
Tumor PD-L1	65%	ND	ND	30%	0%	0%	1%
Metastases at diagnosis	Other LN	Abdominal LN, other LN	Cervical LN, other LN	Other LN	No	Other LN, other organs	Cervical LN, Other LN
Time of receiving nivolumab (months)	70.8	67.6	55.4	46.9	47.2	10.3	29.2
OS (months)	71.3	68.1	67.4	66.6	52.2	41.2	40.3
Best overall response (central review)	Complete response	Complete response	Not assessable	Complete response	Partial response	Partial response	Stable disease
Subsequent therapy	Yes*	Yes*	No	No	No	Yes	Yes

*Commercially available nivolumab was used after approval

*ECOG PS* Eastern Cooperative Oncology Group Performance Status, *LN* lymph node, *ND* not determined, *OS* overall survival

### Safety

The AEs observed in ≥ 5% of the study population are shown in Supplementary Table 3. Of 65 patients, 56 (86.2%) exhibited AEs of any grade, and 21 (32.3%) had AEs of grade 3 or 4. The most common AEs were diarrhea, pneumonia, and decreased appetite. Other notable AEs included abnormal hepatic function of any grade and of grade 3 or 4 found in 4 (6.2%) and 2 (3.1%) patients, respectively. Pneumonia was the only grade 3 or 4 AE reported in ≥ 3 patients. Additionally, 41 (63.1%) patients showed treatment-related AEs, and 13 (20.0%) had grade 3 or 4. The most common treatment-related AEs were diarrhea and rash. Nonetheless, no treatment-related AEs led to death.

### Comparison of efficacy in patients with and without select AEs

**Fig. 3:**
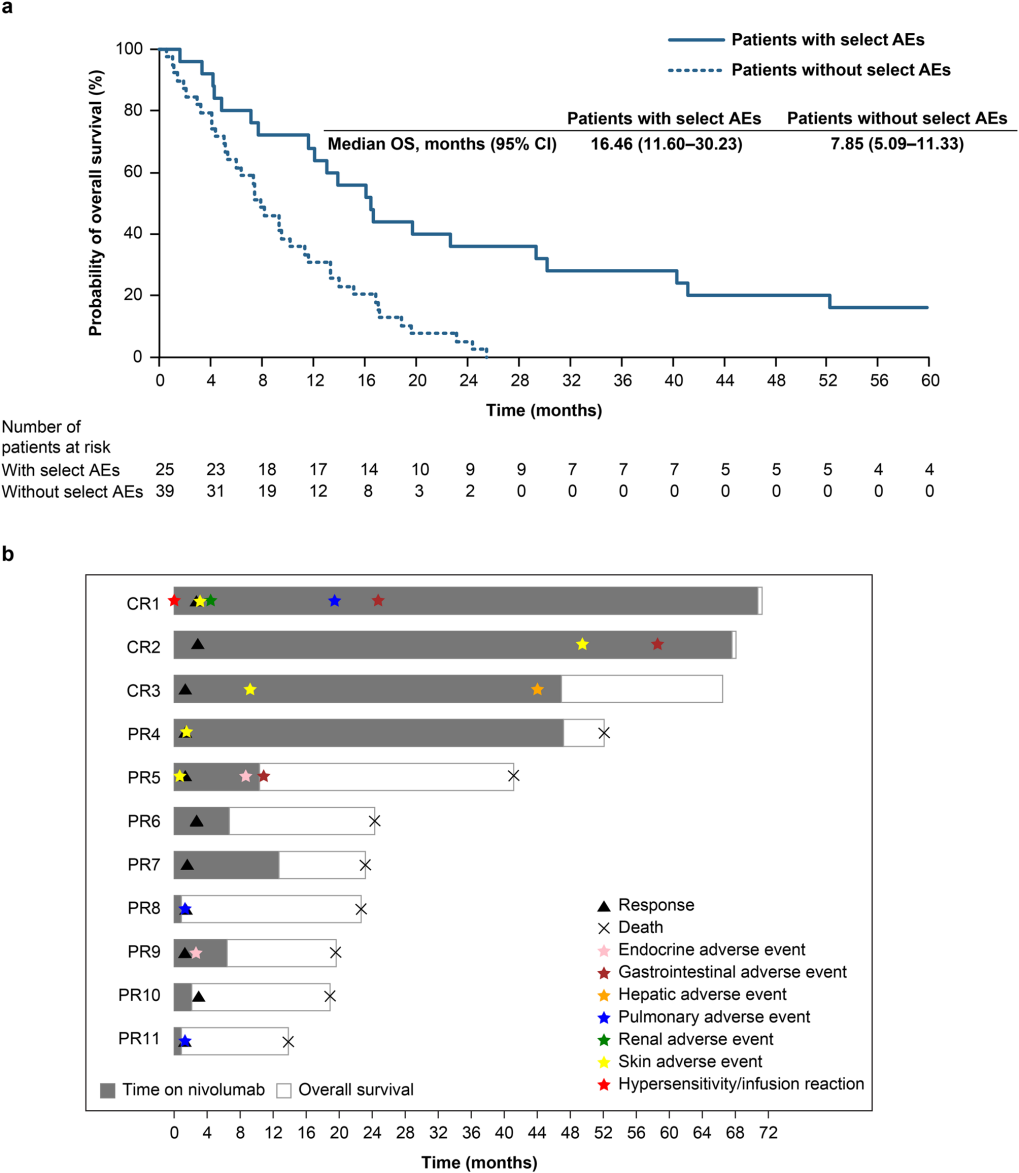
Relationships between select AEs and overall survival or response. **a** The Kaplan–Meier curves shows the overall survival (OS) in patients with and without select adverse events (AEs) are shown. **b** The swimmer plot shows the timing of the response and the first onsets of each category of the treatment-related adverse events in patients with complete response (CR) or partial response (PR) in the nivolumab group

In Kaplan–Meier analysis, patients experiencing select AEs during the whole study period tended to have better OS than those without (Fig. 3a). Among 11 patients who achieved CR or PR, eight exhibited select AEs. One responder exhibited select AEs before response to nivolumab, whereas others exhibited select AEs at the similar timing of or after the response (Fig. 3b).

## Discussion

This analysis in ATTRACTION-1 with a follow-up of > 5 years demonstrated that nivolumab monotherapy has long-term efficacy and safety for patients with chemotherapy-refractory or -intolerant esophageal cancer. Seven and four patients survived for 3 and 5 years, respectively, while the ORR, median OS, and median PFS remained unchanged at 2 years of follow-up [16]. No new safety signals were identified.

The Kaplan–Meier curves of PFS almost plateaued at 1-year follow-up, and 4 out of 5 patients without disease progression at 2-year follow-up remained progression-free for more than 5 years after the first dose of nivolumab. Furthermore, a 5-year survivor had received no subsequent therapy for more than a year; thus, nivolumab could persistently suppress esophageal cancer progression. Five-year survivors without disease progression were also observed in other tumors treated with nivolumab [10, 12].

This report has provided the longest and first 5-year efficacy and safety data of nivolumab for esophageal cancer. Here, two patients were treated with nivolumab for more than 5 years, suggesting that nivolumab monotherapy has long-term tolerability for esophageal cancer. The incidence of treatment-related AEs of grade 3 or 4 during 5-year treatment with nivolumab in patients with esophageal cancer was 20%, which is consistent with those observed in patients with malignant melanoma, non-small cell lung cancer, and renal cell carcinoma (16–23%) [6–11]. The overall incidence of AEs is similar to that in the primary analysis of ATTRACTION-1 with a median follow-up of 10.8 months [14]; hence, most AEs tended to appear within a year of nivolumab treatment. The relatively early onset of AEs related to nivolumab treatment has also been observed in other cancers, including malignant melanoma, non-small cell lung cancer, and gastric cancer [12, 17, 18]. The current study observed that 5 and 2 patients manifested pneumonia as an AE of grade 3–4 and a treatment-related AE of grade 3–4, respectively, after the primary analysis. In addition, 5-year survivors with CR experienced select AEs 4–5 years after the initial dose of nivolumab. Therefore, relatively late-onset AEs should also be carefully monitored.

In this study, patients achieving objective responses accounted for most of the long-term survivors. Additionally, patients exhibiting select AEs tended to survive longer than those without. Thus, even if the patients do not initially respond well to nivolumab, select AEs should be appropriately managed to expect for a long-term survival. Experience of select AEs could be a possible predictor of objective responses and long-term survival [19–21]. Consistent with our study, most patients achieving CR or PR experienced select AEs. However, some patients without select AEs also achieved an objective response. Furthermore, experience of select AEs and objective responses had no apparent chronological orders, as shown in head and neck cancer [22]. Some patients exhibited select AEs before an objective response, while others responded first and then exhibited select AEs. Thus, absence of select AEs should not be an indication to determine the discontinuation of nivolumab treatment.

We acknowledge some limitations in ATTRACTION-1. The single-arm study did not have any comparator groups. Only Japanese patients with ESCC were enrolled. Considering the small sample size, interpretations of subgroup analyses were limited. Long-term survivors may further be characterized in the large-scale phase III ATTRACTION-3 study.

## Conclusion

This analysis represented the longest follow-up of patients with advanced ESCC treated with nivolumab. These patients survived longer through nivolumab treatment, and no new safety signals were identified during the 5-year follow-up.

## Supplementary Information

Below is the link to the electronic supplementary material.

Supplementary file1 (DOCX 53 KB)
