# Supplementary material for: Five-year follow-up of nivolumab treatment in Japanese patients with esophageal squamous-cell carcinoma (ATTRACTION-1/ONO-4538-07)
Source: Esophagus. 2021 May 16;18(4):835–43. doi: 10.1007/s10388-021-00850-0 (PMC8387268; doi:10.1007/s10388-021-00850-0)
Supplement: Supplementary file 1 — Supplementary file1 (DOCX 53 KB) [file 10388_2021_850_MOESM1_ESM.docx]

**Five-year follow-up of nivolumab treatment in Japanese patients with esophageal squamous-cell carcinoma (ATTRACTION-1/ONO-4538-07)**

Taroh Satoh^1,*^, Ken Kato^2^, Takashi Ura^3,a^, Yasuo Hamamoto^4^, Takashi Kojima^5^, Takahiro Tsushima^6^, Shuichi Hironaka^7,b^, Hiroki Hara^8^, Satoru Iwasa^2^, Kei Muro^3^, Hirofumi Yasui^6^, Keiko Minashi^7^, Kensei Yamaguchi^8,c^, Atsushi Ohtsu^9^, Yuichiro Doki^10^, Yasuhiro Matsumura^11^, Yuko Kitagawa^12^

^1^Frontier Science for Cancer and Chemotherapy, Osaka University Graduate School of Medicine, Suita, Japan; ^2^Department of Head and Neck, Esophageal Medical Oncology / Department of Gastrointestinal Medical Oncology, National Cancer Center Hospital, Tokyo, Japan; ^3^Department of Clinical Oncology, Aichi Cancer Center Hospital, Nagoya, Japan; ^4^Keio Cancer Center, Keio University School of Medicine, Tokyo, Japan; ^5^Department of Gastroenterology and Gastrointestinal Oncology, National Cancer Center Hospital East, Kashiwa, Japan; ^6^Division of Gastrointestinal Oncology, Shizuoka Cancer Center, Shizuoka, Japan; ^7^Clinical Trial Promotion Department, Chiba Cancer Center, Chiba, Japan; ^8^Department of Gastroenterology, Saitama Cancer Center, Saitama, Japan; ^9^National Cancer Center Hospital East, Kashiwa, Japan; ^10^Department of Gastroenterological Surgery, Osaka University Graduate School of Medicine, Osaka, Japan; ^11^Department of Oncology, ONO Pharmaceutical Co., Ltd., Osaka, Japan; ^12^Department of Surgery, Keio University School of Medicine, Tokyo, Japan.

^a^Current address: Department of Clinical Oncology, National Hospital Organization Kyoto Medical Center, Kyoto, Japan

^b^Current address: Department of Medical Oncology and Hematology, Oita University Faculty of Medicine, Oita, Japan

^c^Current address: Department of Gastroenterological Chemotherapy, The Cancer Institute Hospital of Japanese Foundation for Cancer Research, Tokyo, Japan

*Corresponding author: Taroh Satoh, MD, PhD

Department of Frontier Science for Cancer and Chemotherapy, Osaka University Graduate School of Medicine, E21-19, 2-2, Yamadaoka, Suita, Osaka, 565-0871 Japan

Phone: +81-6-6879-2641

FAX: +81-6-6879-2639

E-mail: taroh@cfs.med.osaka-u.ac.jp

**Supplementary Table 1** Select adverse events

| Category | Subcategory | Preferred terms |
| --- | --- | --- |
| Endocrine Adverse Events | Adrenal Disorder | Adrenal insufficiency  Adrenal suppression  Adrenocortical insufficiency acute  Blood corticotrophin decreased  Blood corticotrophin increased  Hypothalamic pituitary adrenal axis suppression  Primary adrenal insufficiency  Secondary adrenocortical insufficiency |
|  | Diabetes | Diabetes mellitus  Diabetic ketoacidosis  Diabetic ketosis  Fulminant type 1 diabetes mellitus  Latent autoimmune diabetes in adults  Type 1 diabetes mellitus |
|  | Pituitary Disorder | Hypophysitis  Hypopituitarism  Lymphocytic hypophysitis |
|  | Thyroid Disorder | Atrophic thyroiditis  Autoimmune hypothyroidism  Autoimmune thyroid disorder  Autoimmune thyroiditis  Basedow’s disease  Blood thyroid stimulating hormone decreased  Blood thyroid stimulating hormone increased  Hyperthyroidism  Hypothyroidism  Primary hyperthyroidism  Primary hypothyroidism  Silent thyroiditis  Thyroid function test abnormal  Thyroid hormones decreased  Thyroid hormones increased  Thyroiditis  Thyroiditis acute  Thyroxine decreased  Thyroxine free decreased  Thyroxine free increased  Thyroxine increased  Tri-iodothyronine uptake increased |
| Gastrointestinal Adverse Events |  | Autoimmune colitis  Autoimmune enteropathy  Colitis  Colitis ulcerative  Diarrhea  Duodenal perforation  Enteritis  Enterocolitis  Enterocolitis hemorrhagic  Frequent bowel movements  Gastrointestinal perforation  Lower gastrointestinal perforation  Upper gastrointestinal perforation |
| Hepatic Adverse Events |  | Acute hepatic failure  Acute on chronic liver failure  Alanine aminotransferase increased  Aspartate aminotransferase increased  Autoimmune hepatitis  Bilirubin conjugated increased  Blood alkaline phosphatase increased  Blood bilirubin increased  Drug-induced liver injury  Gamma-glutamyltransferase increased  Hepatic enzyme increased  Hepatic failure  Hepatitis  Hepatitis acute  Hepatotoxicity  Hyperbilirubinemia  Immune-mediated hepatitis  Liver disorder  Liver function test abnormal  Liver function test increased  Liver injury  Transaminases increased |
| Hypersensitivity/  Infusion Reaction |  | Anaphylactic reaction  Anaphylactic shock  Bronchospasm  Hypersensitivity  Infusion related reaction |
| Pulmonary Adverse Events |  | Acute respiratory distress syndrome  Acute respiratory failure  Idiopathic interstitial pneumonia  Interstitial lung disease  Lung infiltration  Pneumonitis |
| Renal Adverse Events |  | Acute kidney injury  Autoimmune nephritis  Blood creatinine increased  Blood urea increased  Creatinine renal clearance decreased  Hypercreatininaemia  Nephritis  Nephritis allergic  Paraneoplastic glomerulonephritis  Renal failure  Renal tubular necrosis  Tubulointerstitial nephritis  Urine output decreased |
| Skin Adverse Events |  | Autoimmune dermatitis  Blister  Dermatitis  Dermatitis allergic  Dermatitis exfoliative  Drug eruption  Eczema  Erythema  Erythema multiforme  Exfoliative rash  Fixed eruption  Nodular rash  Palmar-plantar erythrodysesthesia syndrome  Pemphigoid  Pemphigus  Photosensitivity reaction  Pruritus  Pruritus allergic  Pruritus generalized  Psoriasis  Rash  Rash erythematous  Rash generalized  Rash macular  Rash maculo-papular  Rash morbilliform  Rash papular  Rash pruritic  Rash vesicular  Skin exfoliation  Skin hypopigmentation  Skin irritation  Stevens-Johnson syndrome  Toxic epidermal necrolysis  Toxic skin eruption  Urticaria  Vitiligo |

**Supplementary Table 2** Best overall response (N = 64)

|  | **Central review** | **Investigator assessment** |
| --- | --- | --- |
| **Objective response rate—% (95% CI)** | 17.2 (9.9, 28.2) | 21.9 (13.5, 33.4) |
| **Best overall response—n (%)** |  |  |
| **Complete response** | 3 (4.7) | 2 (3.1) |
| **Partial response** | 8 (12.5) | 12 (18.8) |
| **Stable disease** | 16 (25.0) | 20 (31.3) |
| **Progressive disease** | 29 (45.3) | 29 (45.3) |
| **Not assessable** | 8^a^ (12.5) | 1 (1.6) |
| **Time to initial response—median (range)** | 1.4 months (1.4, 3.0) | ND |
| **Duration of response—median (95% CI)** | 11.2 months (3.0, NR) | ND |

*CI*, confidence interval; *ND*, not determined; *NR*, not reached.

^a^Patients without target lesions were included.

**Supplementary** **Table 3** Adverse events observed in ≥5% patients (N = 65)

|  | **Adverse events** | | **Treatment-related adverse events** | |
| --- | --- | --- | --- | --- |
|  | **Any grade** | **Grade 3 or 4** | **Any grade** | **Grade 3 or 4** |
| Any adverse events | 56 (86.2) | 21 (32.3) | 41 (63.1) | 13 (20.0) |
| Diarrhea | 15 (23.1) | 0 | 11 (16.9) | 0 |
| Pneumonia | 14 (21.5) | 6 (9.2) | 5 (7.7) | 2 (3.1) |
| Decreased appetite | 12 (18.5) | 2 (3.1) | 6 (9.2) | 2 (3.1) |
| Rash | 9 (13.8) | 0 | 7 (10.8) | 0 |
| Cough | 8 (12.3) | 0 | 1 (1.5) | 0 |
| Nasopharyngitis | 8 (12.3) | 0 | 0 | 0 |
| Fatigue | 7 (10.8) | 1 (1.5) | 5 (7.7) | 1 (1.5) |
| Pruritus | 7 (10.8) | 0 | 6 (9.2) | 0 |
| Malaise | 7 (10.8) | 0 | 3 (4.6) | 0 |
| Constipation | 7 (10.8) | 0 | 1 (1.5) | 0 |
| Nausea | 6 (9.2) | 0 | 3 (4.6) | 0 |
| Vomiting | 6 (9.2) | 0 | 2 (3.1) | 0 |
| Back pain | 6 (9.2) | 0 | 0 | 0 |
| Fever | 5 (7.7) | 0 | 3 (4.6) | 0 |
| Blood creatine phosphokinase increased | 4 (6.2) | 2 (3.1) | 4 (6.2) | 2 (3.1) |
| Hepatic function abnormal | 4 (6.2) | 2 (3.1) | 2 (3.1) | 1 (1.5) |
| Infusion related reaction | 4 (6.2) | 0 | 4 (6.2) | 0 |
| Dysgeusia | 4 (6.2) | 0 | 3 (4.6) | 0 |
| Hypothyroidism | 4 (6.2) | 0 | 3 (4.6) | 0 |
| Edema | 4 (6.2) | 0 | 0 | 0 |
| Pain | 4 (6.2) | 0 | 0 | 0 |
| Upper respiratory tract infection | 4 (6.2) | 0 | 0 | 0 |
